# EGFR-induced phosphorylation of type Iγ phosphatidylinositol phosphate kinase promotes pancreatic cancer progression

**DOI:** 10.18632/oncotarget.16730

**Published:** 2017-03-31

**Authors:** Chunhua Chen, Xiangling Wang, Juemin Fang, Junli Xue, Xunhao Xiong, Yan Huang, Jinghua Hu, Kun Ling

**Affiliations:** ^1^Department of Biochemistry and Molecular Biology, Mayo Clinic, Rochester, Minnesota, USA; ^2^Shanghai Tenth People’s Hospital, Tongji University, Shanghai, China; ^3^Shanghai East Hospital, Tongji University, Shanghai, China; ^*^These authors have contributed equally to this work

**Keywords:** pancreatic cancer, type Iγ phosphatidylinositol phosphate kinase, EGFR, tyrosine phosphorylation, metastasis

## Abstract

Pancreatic cancer is one of the deadliest malignancies and effective treatment has always been lacking. In current study, we investigated how the type Iγ phosphatidylinositol phosphate kinase (PIPKIγ) participates in the progression of pancreatic ductal adenocarcinoma (PDAC) for novel therapeutic potentials against this lethal disease. We found that PIPKIγ is up-regulated in all tested PDAC cell lines. The growth factor (including EGFR)-induced tyrosine phosphorylation of PIPKIγ is significantly elevated in *in situ* and metastatic PDAC tissues. Loss of PIPKIγ inhibits the aggressiveness of PDAC cells by restraining the activities of AKT and STAT3, as well as MT1-MMP expression. Therefore when planted into the pancreas of nude mice, PIPKIγ-depleted PDAC cells exhibits substantially repressed tumor growth and metastasis comparing to control PDAC cells. Results from further studies showed that the phosphorylation-deficient PIPKIγ mutant, unlike its wild-type counterpart, cannot rescue PDAC progression inhibited by PIPKIγ depletion. These findings indicate that PIPKIγ, functioning downstream of EGFR signaling, is critical to the progression of PDAC, and suggest that PIPKIγ is potentially a valuable therapeutic target for PDAC treatment.

## INTRODUCTION

Pancreatic ductal adenocarcinoma (PDCA), due to the early onset of local invasion and distant metastasis, is one of the most lethal human malignancies with < 5% 5-year survival rate [1, 2]. Thus, it is essential to understand the mechanisms underlying PDAC metastasis in order to develop better therapeutic approaches. It was shown recently that activation of EGFR is necessary for the progression of pancreatic intraepithelial neoplasia (PanIN) to aggressive PDAC [3]. However, PDAC patients respond poorly to EGFR-targeting drugs [4]. One possible reason is that other signaling pathway, such as hepatocyte growth factor (HGF) via its receptor c-Met, shares common downstream signaling cascades as EGF and compensates EGFR inhibition. In this context, in-depth understanding of EGF pathway in PDAC progression is essential for the development of novel therapeutic strategies.

Type Iγ phosphatidylinositol phosphate kinase (PIPKIγ), upon being phosphorylated by EGF and/or HGF stimulation [5, 6], modulates subcellular production of phosphatidylinositol-4,5-bisphosphate (PtdIns(4,5)P_2_) and regulates a variety of cellular processes important for cancer progression, such as cell survival, cell cycle progression, focal adhesion dynamics, vesicular trafficking and actin cytoskeleton reorganization [7–11]. Indeed, elevated PIPKIγ activity, reported in hepatocellular carcinoma [12] and breast cancer and inversely correlates with the survival of breast cancer patients [5, 13]. Moreover, depletion of PIPKIγ impaired the migration and invasion of breast and colon cancer cells [5, 14]. Considering the vital role of EGFR in the development of PDAC [3], it is important to investigate the precise function of PIPKIγ in this deadly disease.

Here we report that PIPKIγ is upregulated and substantially phosphorylated in PDAC cells and patient tissues. Loss of PIPKIγ compromised the proliferation, migration, matrix degradation, and invasion of PDAC by inhibiting the activation of AKT and STAT3, as well as the production of membrane type 1-matrix metalloproteinase 1 (MT1-MMP or MMP14). Consequently, the growth and metastasis of PIPKIγ-depleted PDAC cells were remarkably attenuated comparing to control cells when orthotopically transplanted into mice. Moreover, the ectopic expression of phosphorylation-deficient PIPKIγ mutant cannot rescue the aggressive *in vitro* and *in vivo* behaviors of PIPKIγ-depleted PDAC tumor cells, whereas its wild-type counterpart can. These findings define PIPKIγ as an important component of EGFR pathway during the development of aggressive PDAC and suggest PIPKIγ as a novel therapeutic target for the clinical management of PDAC.

## RESULTS

### PIPKIγ expression is upregulated in PDACs

**Figure 1:**
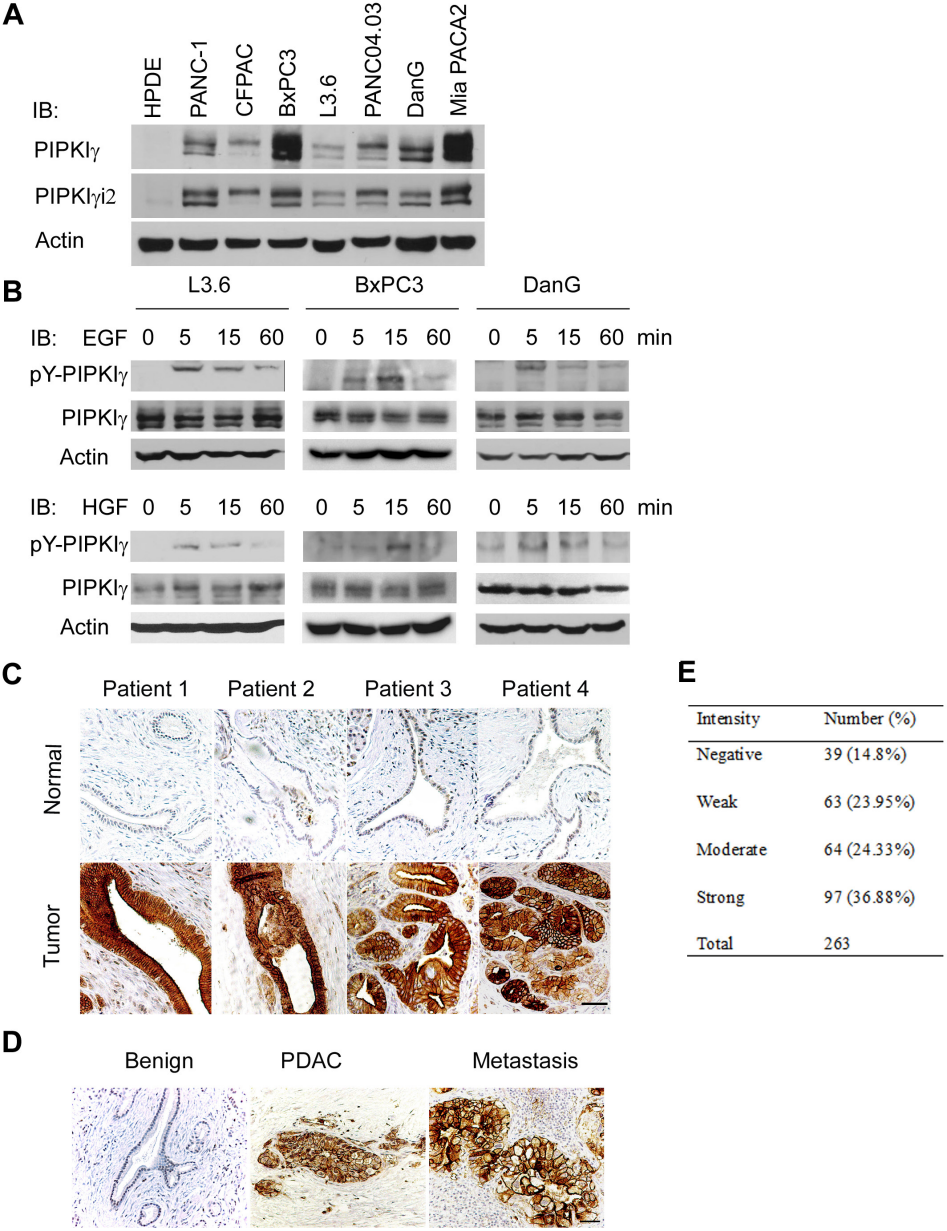
PIPKIγ is upregulated in human pancreatic ductal carcinoma. **(A)** PIPKIγ expression is increased in cultured PDAC cells. Indicated normal human pancreatic ductal epithelial cells (HPDE) and different types of malignant PDAC cells were collected to make cell lysates for immunoblotting analyses with anti-PIPKIγ antibody. **(B)** PIPKIγ is phosphorylated at Y639 responding to EGF or HGF stimulation. Three different types of PDAC cells were serum starved overnight and treated with 10 ng/mL EGF or HGF for indicated time. Then cell lysates were prepared for immunoblotting with antibodies against total (pan-PIPKIγ) or Y639-phosphorylated (pY-PIPKIγ) PIPKIγ. **(C and D)** Phosphorylation level of PIPKIγ is dramatically increased in PDAC lesions. **(C)** pY-PIPKIγ antibody was used to stain human PDAC tissues (lower panels, tumor) and matched adjacent non-tumor tissues (upper panels, normal). Staining results from 263 patients were summarized in right panel. **(D)** Metastatic PDAC lesions also exhibit high level of pY639-phosphorylated PIPKIγ. Representative images of immunohistochemistry staining using pY-PIPKIγ antibody in benign, PDAC and lymphoid node metastases from the same patient were shown. Scale bar, 50μm.

PIPKIγ, by generating PtdIns(4,5)P_2_, regulates multiple cellular processes including cell proliferation and survival, cell adhesion and migration, and membrane and protein transport. Among the five known alternative splicing isoforms of PIPKIγ [15], the isoform 2 (PIPKIγi2) specifically targets to focal adhesions and regulates cell migration [6, 9, 16], suggesting a potential of participating in tumor metastasis. To investigate the role of PIPKIγ in pancreatic cancer, we first examined the expression of total PIPKIγ and PIPKIγi2 in human PDAC cell lines. Comparing to the normal human pancreatic ductal epithelial HPDE cells, total PIPKIγ levels are markedly increased in all seven tested PDAC lines with a remarkable elevation in BxPC3 and Mia PACA2 (Figure 1A). Protein level of PIPKIγi2 is also significantly upregulated in these PDAC cells with a similar trend as that of the total PIPKIγ (Figure 1A).

We showed previously that PIPKIγ could be phosphorylated by EGFR at Y649, which is critical for the directional migration and metastasis of breast tumor cells [5, 6]. To determine whether this is also true in pancreatic tumor, we treated three different types of PDAC cells (L3.6, BxPC3, and DanG) with EGF, and then analyzed the cell lysates using an antibody specifically recognizing Y639-phosphorylated (pY639) PIPKIγ [5]. As shown in Figure 1B (upper panels), EGF stimulation led to PIPKIγ phosphorylation at Y639 in all three types of cells and the phosphorylation level of PIPKIγ peaked at 5 minutes upon EGF treatment. Interestingly, HGF also caused PIPKIγ phosphorylation in these cells (Figure 1B, lower panels). It was shown lately that blockade of EGF/EGFR attenuates pancreatic tumorigenesis induced by KRAS^G12D^ or pancreatitis [3], which supports the essential role of EGF signaling in PDAC. Recent studies also indicate that the signaling axis of HGF and its receptor c-Met plays an important role in the interaction between PDAC-associated microenvironment and PDAC, therefore promoting desmoplasia and chemoresistance in pancreatic cancer [17]. In this context, our results suggest that PIPKIγ might participate in the progressin of PDAC from multiple aspects as an important signaling cascade downstream of both EGFR and c-Met.

To investigate this possibility, we analyzed the levels of phosphorylated PIPKIγ in PDAC patients using the pY639-PIPKIγ antibody. Although the paired adjacent normal tissues universally appear negative, the PDAC lesions exhibit remarkably strong staining of pY639-PIPKIγ (Figure 1C). Moreover, high level of PIPKIγ phosphorylation was also observed in the matched lymph node metastatic tissues (Figure 1D). In comparison, staining of pan-PIPKIγ antibody that reflects the total protein levels of PIPKIγ is slightly elevated in cancerous tissues (Supplementary Figure 1). These observations suggest that the increase of Y639 phosphorylation, rather than the protein level, of PIPKIγ is more important for PDAC progression. This was further inspected by tissue microarray containing 263 PDAC patients. As shown in Figure 1E, 85% of the patients exhibit positive PIPKIγ phosphorylation and ~ 37% of them show strong phosphorylation, indicating that PIPKIγ plays an important role in PDAC, which provides rationale for our further investigation for the role of PIPKIγ in PDAC progression.

### PIPKIγ is required for the activation of signaling cascades downstream of EGFR in PDAC cells

**Figure 2:**
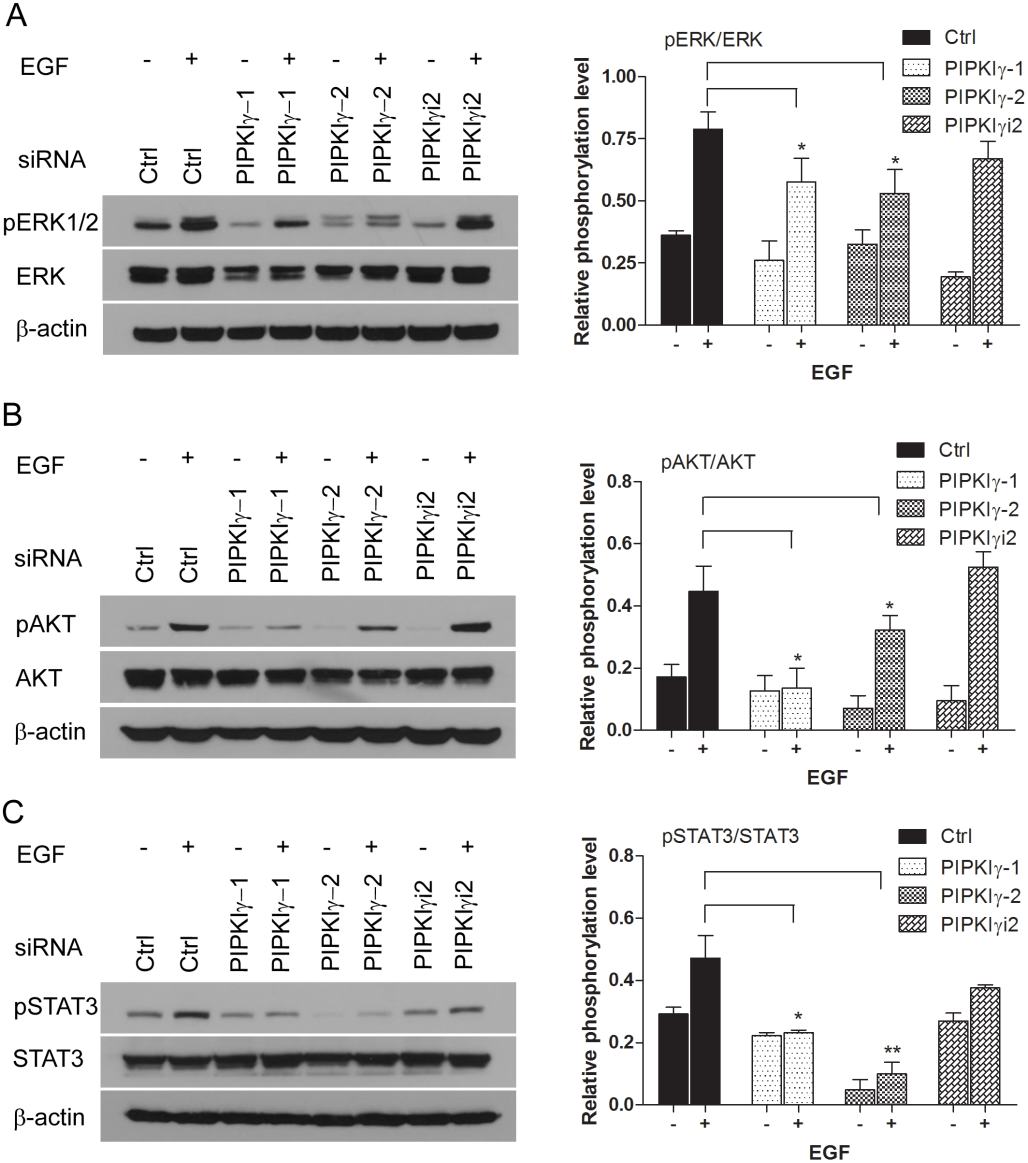
PIPKIγ is required for EGF-induced activation of MAPK, PI3K/AKT and STAT3 pathways. The expression of PIPKIγ was silenced using three different siRNA sequences in DanG cells for 48 hours. With or without 15 minutes EGF (10 ng/mL) stimulation after overnight serum starvation, cells were subjected to immunoblotting using indicated antibodies to examine the activation of ERK1/2 **(A)**, AKT **(B)**, or STAT3 **(C)**. pERK1/2, phosphorylated ERK1/2; ERK, total ERK1/2; pAKT, phosphorylated AKT; AKT, total AKT; p-STAT3, phosphorylated STAT3; STAT3, total STAT3. ImageJ was used to measure the intensity of each band. Intensities of phosphorylated ERK1/2, AKT or STAT3 bands were normalized against ERK1/2, AKT, or STAT3, respectively to represent the relative activation level of these kinases. Results from three independent experiments were statistically analyzed and plotted as panels shown at the right. *, *p*<0.05; **, *p*<0.01.

To understand how PIPKIγ participates in PDAC, we designed multiple specific siRNA sequences that can knock down > 90% of all PIPKIγ splicing isoforms (pan-PIPKIγ) from PDAC cells (Supplementary Figure 2A). Although we only found one PIPKIγi2-specific siRNA sequence due to the similarity among PIPKIγ isoforms, it is efficient enough for PIPKIγi2 depletion in these cells (Supplementary Figure 2A). Using these tools, we first investigated the functional role of PIPKIγ in PDAC cells by examining the pathways that are often hyper-activated in PDACs, such as AKT and MAPK pathways [18]. In cells cultured in complete medium with 10% serum, depletion of all PIPKIγ isoforms or PIPKIγi2 alone has no effect on the basal activity level of MAPK or AKT (Supplementary Figure 3A-3B). However under serum-deficient culturing condition, EGF-induced MAPK and AKT activations were both significantly decreased in PIPKIγ-depleted L3.6 cells comparing to control cells (Figure 2A and 2B). These results suggest that the growth/survival signal pathways in cells lacking PIPKIγ enzymes are less responsive to growth factors thus can be less compatible with nutrition-lacking environment, which often is the condition of microenvironment associated with fast-growing tumors. Specific depletion of PIPKIγi2 had no effect on EGF-induced phosphorylation of MAPK (Figure 2A) or AKT (Figure 2B), suggesting that another PIPKIγ isoform but not PIPKIγi2 is required for the EGFR-stimulated proliferation and growth.

In addition to MAPK and AKT, signal transducer and activator of transcription 3 (STAT3) is another key regulator of cell survival and proliferation, as well as stem cell self-renewal in variant types of cancers [19, 20]. Constitutive activation of STAT3 is frequently detected in PDAC patients and associated with a poor prognosis [21]. Similar as MAPK and AKT activation, basal STAT3 activity is not changed by depleting any isoforms of PIPKIγ comparing to control cells, whereas STAT3 phosphorylation stimulated by EGF was inhibited in pan-PIPKIγ depleted L3.6 cells (Figure 2C), suggesting the requirement of PIPKIγ in growth factor-induced activation of JAK2/STAT3 pathway. Depletion of PIPKIγi2 alone also failed to affect STAT3 phosphorylation under any condition, suggesting that other splicing isoforms of PIPKIγ such as PIPKIγi1 likely involves in STAT3 regulation in these cells. Similar results were observed in DanG cells when examining the activation of MAPK, AKT or STAT3 upon PIPKIγ depletion (Supplementary Figure 3), which further supports the prerequisite of PIPKIγ to common survival pathways in PDAC cells.

**Figure 3:**
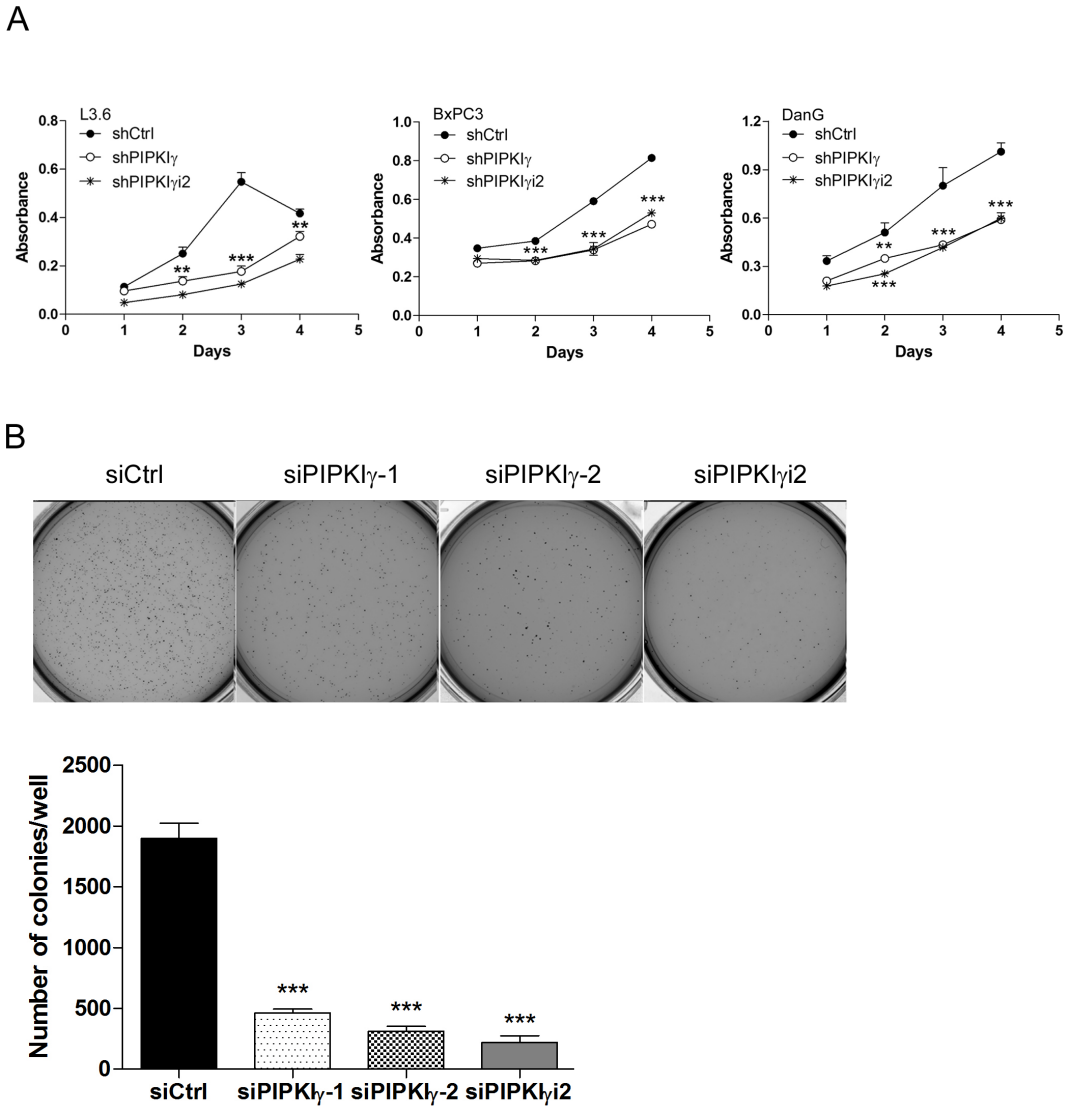
Depletion of PIPKIγ suppressed the proliferation and survival of PDAC cells. Three different types of PDAC cells were infected with lentivirus carrying shRNA sequence for control (shCtrl), all PIPKIγ isoforms (sh IPKIγ), or PIPKIγi2 (shPIPKIγi2) for 48 hrs. Then cells were subjected to MTT assays at indicated time points **(A)**. **(B)** L3.6 cells were transfected with control, PIPKIγ, or PIPKIγi2 siRNA; After 48 hours, soft agar colony formation was analyzed. Indicated L3.6 cells were harvested and suspended in culture medium containing 0.3% agarose, then plated in 6-well plates coated with 0.6% agar in triplicate. 14 days later, cells were stained with MTT and representative images from each group were taken and shown. Colony number in each well was quantified using GelCount software. Results from three independent experiments were statistically analyzed and plotted. ***, p<0.001.

Our results suggested that PIPKIγ is necessary for the phosphorylation/activation of multiple key signaling elements in EGF pathway that are critical for the survival and growth of PDAC cells. To determine the physiological relevance of these observations made at protein levels, we prepared lentivirus-based shRNAs for stable delivery of PIPKIγ depletion in PDAC cells (Supplementary Figure 4) and then analyzed cellular functions. Consistent with the compromised MAPK, AKT and STAT3 activation, the cell proliferation was significantly inhibited in all three different types of PDAC cells when PIPKIγ was depleted (Figure 3A). Results from the soft agar tumor colony formation assay also revealed much less anchorage-independent growth in pan-PIPKIγ-depleted L3.6 cells comparing to control cells (Figure 3B), further suggesting that PIPKIγ-depleted PDAC cells are likely less tumorigenesis. Although depletion of PIPKIγi2 alone showed less effect on MAPK, AKT, or STAT3 activation comparing to what caused by depletion of all PIPKIγ isoforms, it led to a similar level of reduction on cell proliferation (Figure 3A) and anchorage-independent growth (Figure 3B). This suggests that an untested growth/survival pathway that contributes more to these assays might require PIPKIγi2.

### PDAC cells require PIPKIγ for focal adhesion assembly, cell migration, and matrix degradation

**Figure 4:**
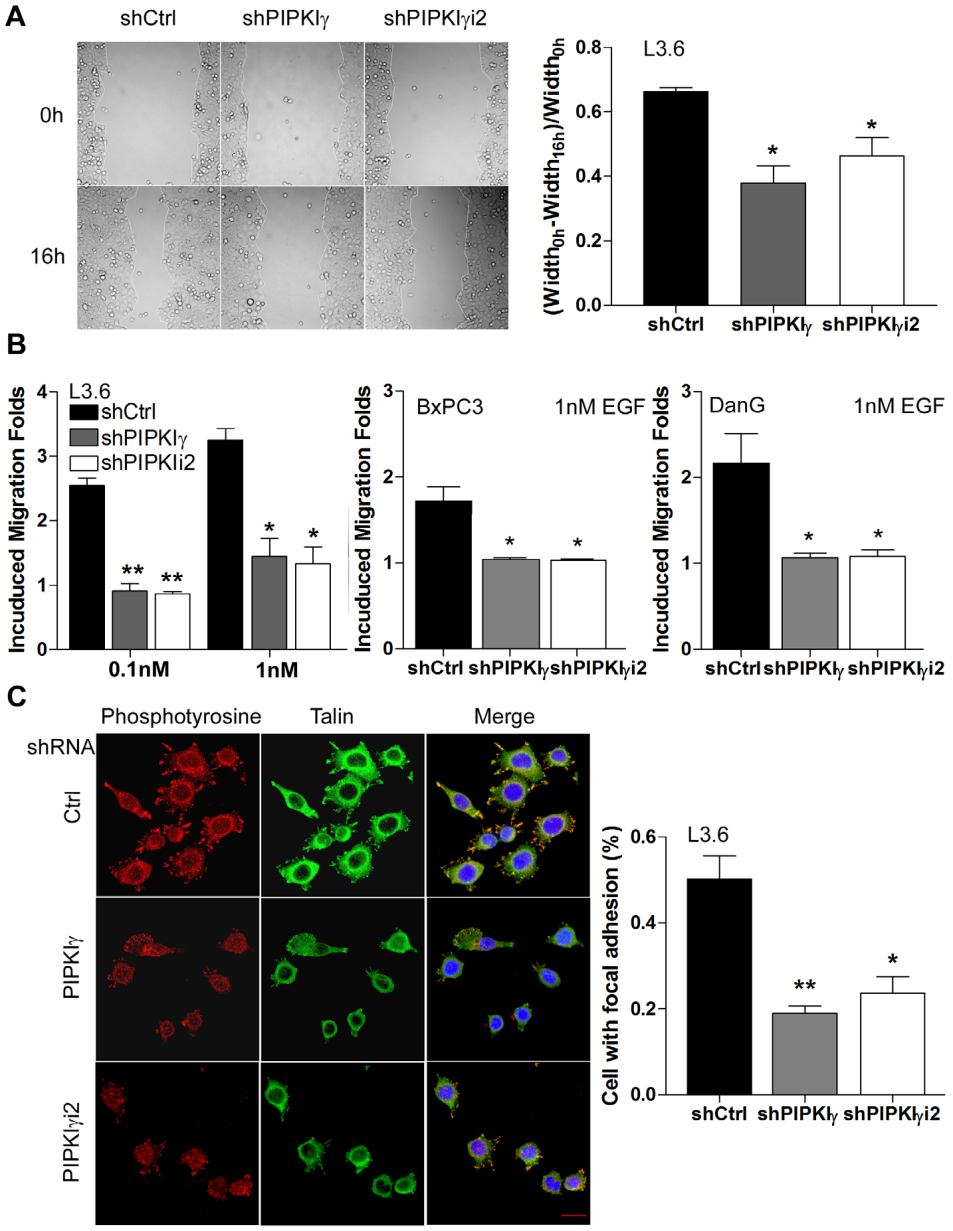
PIPKIγ is necessary for migration of PDAC cells by regulating focal adhesion assembly. L3.6, BxPC3, or DanG cells were treated with lentivirus carrying control (sCtrl), pan-PIPKIγ (shPIPKIγ), or PIPKIγi2-specific (shPIPKIγi2) shRNA for 48 hours, then subjected to wound healing (**A**, L3.6 cells), Boyden Chamber migration assay **(B)**, or immunofluorescence microscopy (**C**, L3.6 cells). **(A)** Representative images of indicated L3.6 cells at 0 and 16 hours in wound healing assay (*left panel*). Quantification of cell motility was represented by measuring the width of the wound (*right panel*). **(B)** Indicated cells were seeded in the upper chamber of Boyden Chamber and incubated for 4-6 hours with indicated concentration of EGF in the lower chamber. **(C)** Control or PIPKIγ-depleted L3.6 cells were plated on tyoe I collagen-coated glass coverslip for 1 hour. Cells were then fixed and stained with antibodies against phosphotyrosine (red) or talin (green) for immunofluorescence microscopy analyses. DAPI (blue) was used to stain the nucleus. Percentage of cells with normal focal adhesions was quantified and plotted. n=100 cells/sample. Scale bar, 20μm. All statistic analyses were performed using results from three independent experiments. *, *p*<0.05; **, *p*<0.01.

In addition to proliferation and survival, the migratory and invasion abilities of cancer cells is critical for the development of tumor metastasis, the primary cause of mortality in most cancers including PDAC. Regulated assembly and turnover of focal adhesions (FAs) are indispensible for growth factors/cytokines-induced directional cell migration, the communication between tumor and tumor-associated microenvironment, and metastasis formation. We and others have shown that PIPKIγi2, which generates local PtdIns(4,5)P_2_ pools to support focal adhesion assembly, is required for EGF-stimulated cell migration in variant types of cells [9, 22–24]. To determine whether PIPKIγ regulates the migration of PDAC cells, we performed *in vitro* cell migration assay including wound healing and Boyden Chamber assays. As shown in Figure 4A, depletion of either pan-PIPKIγ or PIPKIγi2 significantly slowed down the wound-healing rate in L3.6 cells comparing to the control cells treated with scrambled control shRNA. When we determined the EGF-induced directional migration using Boyden Chamber, we observed a much-reduced motility in pan-PIPKIγ or PIPKIγi2 depleted group as compared with the relevant control group of L3.6, BxPC3, or DanG cells (Figure 4B). Consistently, L3.6 cells expressing control shRNA assembled FAs much more efficiently than cells expressing pan-PIPKIγ or PIPKIγi2 shRNA (Figure 4C). These results provide an explanation for the inhibited migratory ability of PIPKIγ-depleted cells and support the requirement of PIPKIγi2 in the migration of PDAC cells, which is consistent with the role of PIPKIγi2 in FA turnover and cell migration as we showed previously in other cell types.

**Figure 5:**
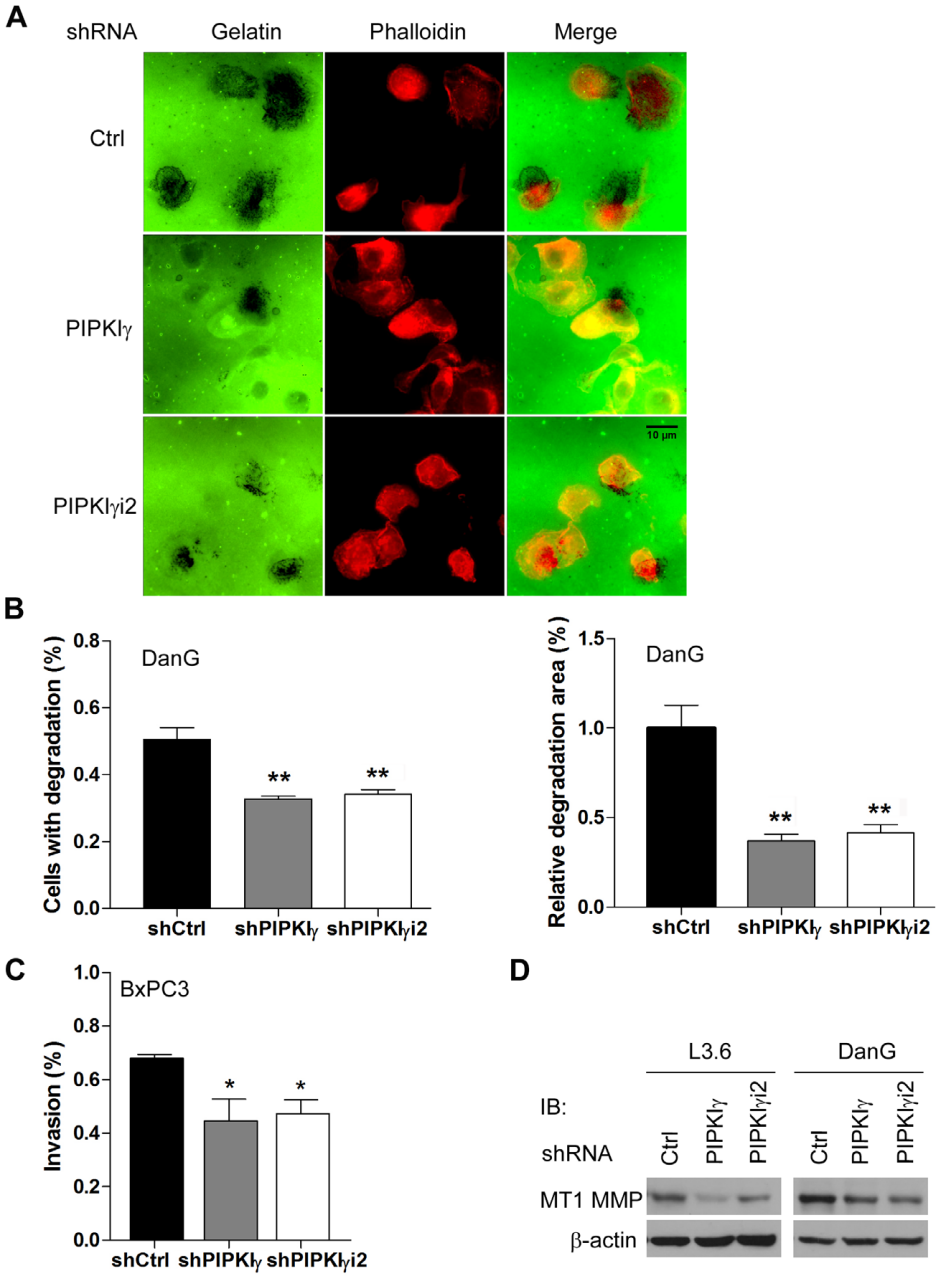
Loss of PIPKIγ led to reduced matrix degradation and invasion in PDAC cells. DanG **(A, B and D)**, L3.6 **(D)** or BxPC3 **(C and D)** cells were transduced by lentivirus expressing control, pan-PIPKIγ or PIPKIγi2 shRNA for 48 hours. **(A)** DanG cells from each group were plated on fluorescent gelatin-coated coverslips (green) for 4 hours, fixed and labele with Alexa Flour-555 conjugated phalloidin (red), and then subjected to fluorescence microscope to analyze gelatin degradation, which appears as black areas beneath the cells. **(B)** Quantification of results obtained in experiments described in **(A)**. Left panel, quantification of cells associated with matrix degradation. n>100 cells. Right panel, quantification of the degradation area normalized against cell number in each field (60x lens). **(C)** Matrigel Transwell chambers were used to perform the *in vitro* invasion assay with BxPC3 cells from indicated group. Invaded cells were quantified following manufacturer’s instruction and plotted. **(B and C)** results from three independent experiments were analyzed. *, *p*<0.05; **, *p*<0.01. **(D)** Protein levels of MT1-MMP were determined in indicated L3.6 and DanG cells by immunoblotting using indicated antibodies.

In addition to cell migration, the ability to degrade the extracellular matrix (ECM) is critical for tumor cells to be invasive and metastatic. To test whether PIPKIγ participates in cell invasion, DanG cells expressing control, pan-PIPKIγ, or PIPKIγi2 shRNA were utilized to perform matrix degradation assay. In this experiment, DanG cells showed a range of matrix degradation patterns including punctate or blotchy areas devoid of fluorescein-gelatin fluorescence. As shown in Figure 5A, the gelatin degradation achieved by pan-PIPKIγ or PIPKIγi2-depleted cells was markedly less comparing to the control cells. We quantified the percentage of cells exercising matrix degradation and the average degradation area for each experimental group. The results (Figure 5B) clearly showed that loss of pan-PIPKIγ or PIPKIγi2 significantly inhibited the matrix degradation in DanG cells. To further confirm the role of PIPKIγ in invasion of pancreatic cancer cells, we also employed the Transwell-based Matrigel invasion assay. As shown in Figure 5C, depletion of pan-PIPKIγ and PIPKIγi2 both compromised the ability of cells to invade into the Matrigel. Our results indicated that pan-PIPKIγ depletion and PIPKIγi2-alone depletion share similar effect on inhibiting PDAC invasion, suggesting that PIPKIγi2 is the main PIPKIγ isoform regulating this process.

MT1-MMP, a membrane-bound matrix metalloproteinase (MMP) localizing to the leading edge of invasive cells, degrades ECM components and activates latent MMP-2 [25–29], therefore has been strongly implicated in metastasis of multiple types of cancers [29]. Recently, it was shown that upregulation of MT1-MMP significantly correlates with a poor prognosis of PDAC patients [30, 31], further supporting the importance of this MMP in PDAC progression. Interestingly, the protein levels of MT1-MMP were significantly lower in L3.6 and DanG cells treated with pan-PIPKIγ or PIPKIγi2 specific shRNA comparing to cells treated by control shRNA (Figure 5D). This piece of result suggests that PIPKIγ isoforms, PIPKIγi2 in particular, is required for the maintenance of MT1-MMP levels, which contributes to the matrix degradation and invasion of PDAC cells.

### Depletion of PIPKIγ suppressed PDAC progression *in vivo*

**Figure 6:**
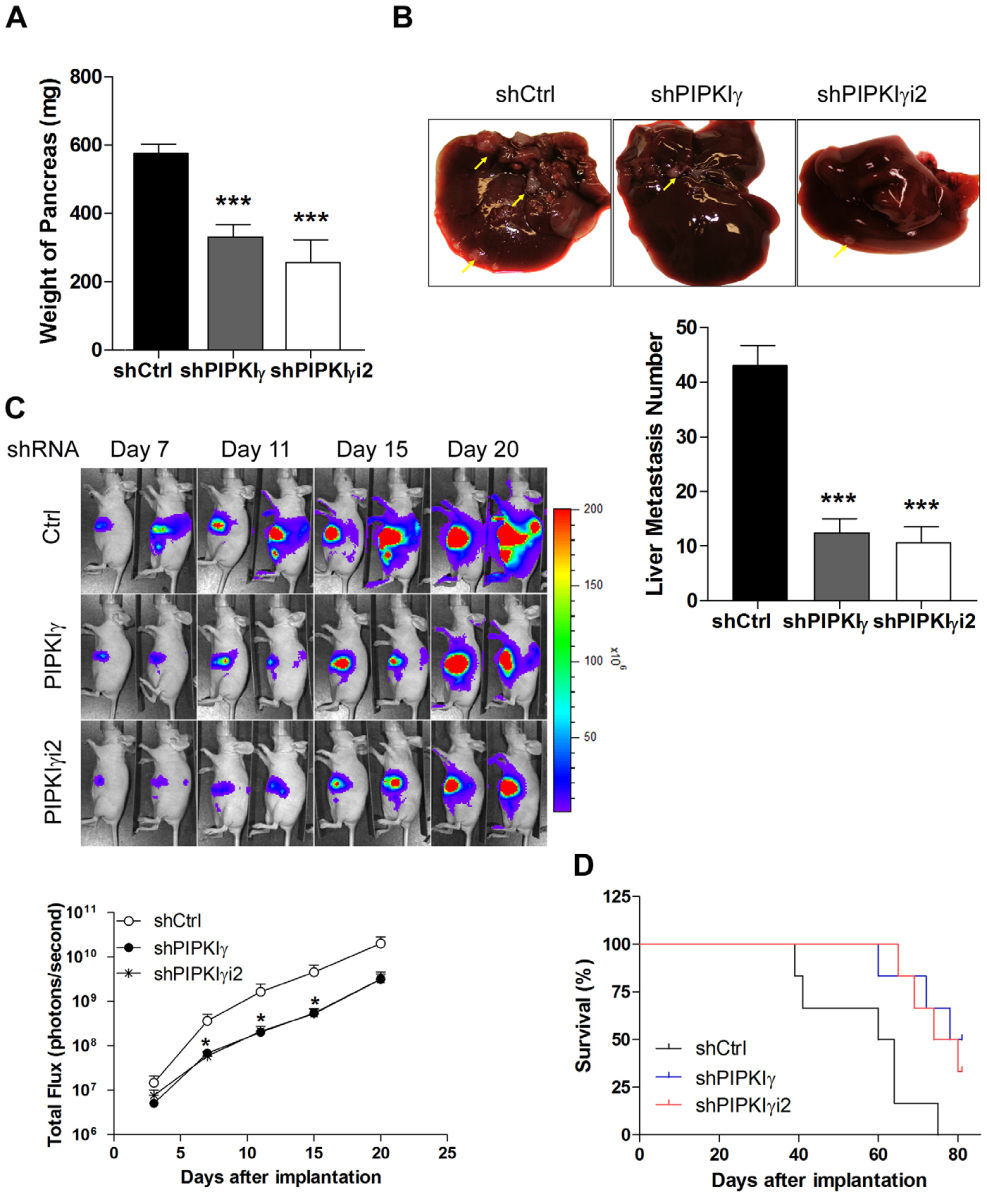
Deficient PIPKIγ suppresses the growth and metastasis of PDAC tumors in mice. L3.6 cells (1.0×10^6^ cells/mouse) stably expressing control, PIPKIγ or PIPKIγi2 shRNA were injected into the pancreas of nude mice. n =10 mice/group. Mice were sacrificed at day 21 after injection. **(A)** Tumor weight was determined and plotted. **(B)** Liver metastasis was analyzed. Upper panels, typical liver images from a representative mouse in indicated group. Lower panel, number of metastatic nodules on liver surface in each animal was counted in each group and plotted. ***, *p*<0.001. **(C)** Representative luminescence images of nude mice injected with luciferase-expressing L3.6 cells expressing control, PIPKIγ or PIPKIγi2 shRNA (5×10^5^ cells/mouse) were showed at indicated time after orthotopic injection (*upper*). Measurements of photons/s/cm2/steridian depicting bioluminescence area of each group were plotted (*lower*). **(D)** Survival (Kaplan-Meier) curve of nude mice injected with control, PIPKIγ or PIPKIγi2-depleted L3.6 cells was monitored. The latter two groups exhibited significantly improved survival comparing to the control group. Log-rank text, *p*<0.0001.

Our current results demonstrated that loss of PIPKIγ isoforms affects the cellular processes of PDAC cells that are critical for cancer progression, such as proliferation, migration and invasion. This raises a possibility that PIPKIγ might be required for the progression of PDAC *in vivo*. To test this, we employed an orthotopic transplantation mouse model where L3.6 cells stably expressing the control, pan-PIPKIγ, or PIPKIγi2 shRNA were inoculated into the pancreata via surgery. Three weeks post-transplantation, we sacrificed the animals and collected and analyzed pancreas and livers. As shown in Figure 6A, the average weight of pancreas in mice carrying pan-PIPKIγ or PIPKIγi2-depleted L3.6 cells was about half of that in the control mice, indicating that the pancreas mass increase caused by tumor growth is inhibited when PIPKIγ is depleted. This is consistent with the results of *in vitro* cell proliferation and anchorage-independent growth assays (Figure 3A and 3B). When evaluating the liver metastasis by counting the number of tumor nodules on the liver surface, we observed ~3-fold less liver metastasis in mice inoculated with pan-PIPKIγ or PIPKIγi2 depleted L3.6 cells comparing to that in animals of the control group (Figure 6B). In the context that metastasis is heavily dependent upon the abilities of a cell to migrate and degrade the ECM, results from our *in vivo* animal model are fully supported by the results shown previously from *in vitro* migration and invasion assays (Figure 4 and Figure 5).

To monitor the tumor progression in live animals, we introduced the lentivirus-mediated stable expression of luciferase into L3.6 cells that carry the control, pan-PIPKIγ, or PIPKIγi2 shRNA to allow real-time bioluminescence imaging. In the first three weeks post inoculation, mice in the control group constantly exhibited stronger luciferase signal than animals in the pan-PIPKIγ or PIPKIγi2 depletion group (Figure 6C). Indeed, quantitative evaluation of bioluminescence intensity showed significantly slower tumor growth in pan-PIPKIγ and PIPKIγi2 depletion group than that in the control group (Figure 6C). Consistently, the L3.6 PDAC tumor carrying mice in the pan-PIPKIγ or PIPKIγi2 depletion groups exhibited substantially improved survival comparing to animals in the control group (Figure 6D), strongly indicating that loss of PIPKIγ is beneficial to PDAC patients. No significant difference on these beneficial effects was observed between the pan-PIPKIγ and PIPKIγi2 depletion, indicating that PIPKIγi2 plays a major role in promoting PDAC progression, likely by mediating cell migration and invasion. We have shown previously that PIPKIγi2 upon phosphorylated by EGFR is important for cell migration [6]. In the context that EGFR is necessary for the development of PDAC [3], our current results suggest that PIPKIγi2 might be a key player in PDAC progression by coordinating multiple cellular processes stimulated by EGF such as cell migration, MT1-MMP expression and invasion.

### PIPKIγi2 regulates PDAC progression downstream of EGFR

**Figure 7:**
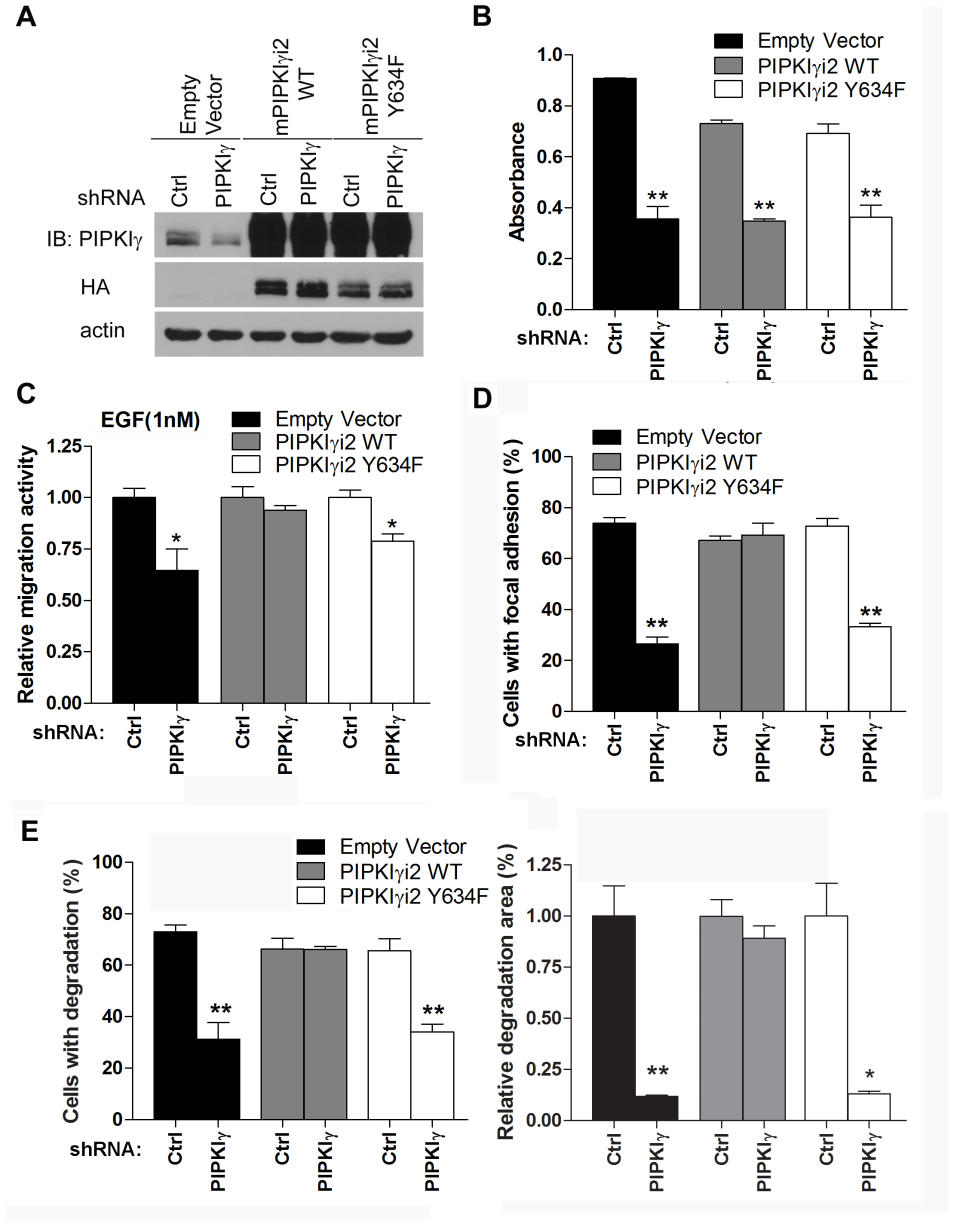
Y639 phosphorylation in PIPKIγ is required for focal adhesion assembly, cell migration, and matrix degradation in PDAC cells. HA-tagged wild type (WT) or Y634F mouse PIPKIγi2 (mPIPKIγi2) was stably expressed in L3.6 or DanG cells by lentivirus-mediated transduction. Then cells were transfected with lentivirus carrying control or hPIPKIγ-specific shRNA to knock down the endogenous hPIPKIγ. **(A)** The expression of HA-tagged wild type (PIPKIγi2 WT) or Y634F (PIPKIγi2 Y634F) mPIPKIγi2 and the depletion of endogenous hPIPKIγ in L3.6 cells were confirmed by immunoblotting using indicated antibodies. **(B)** The proliferation of indicated L3.6 cell lines was determined using MTT assay. mPIPKIγi2 did not restore the *in vitro* proliferation defect caused by depletion of endogenous hPIPKIγ isoforms. **(C-E)** Expression of wild type mPIPKIγi2, but not its Y634 mutated couterpart, rescued the defective cell migration **(C)**, focal adhesion assembly **(D)**, and matrix degradation **(E)** in hPIPKIγ-depleted cells. **(C)** Cells from indicated L3.6 lines were subjected to Boyden Chamber migration assay with 1 nM EGF as attractant. **(D)** L3.6 cells from indicated lines were plated on collagen-coated glass coverslip for 1 hour in complete medium. Cells were then fixed and subjected to indirect immunofluorescence staining with antibodies against phosphotyrosine (green) and talin (red). DAPI (blue) was used to visualize the nucleus. The percentage of cells (n=100 cells/group) with mature focal adhesions was quantified under fluorescence microscope. **(E)** DanG cells expressing indicated shRNA and cDNA were plated on fluorescent gelatin-coated coverslips and subjected to matrix degradation assay. Left panel, quantification of cells associated with matrix degradation. Right panel, quantification of degradation area normalized against cell number. Results from three independent experiments were statistically analyzed and plotted. *, *p*<0.05. **, *p*<0.01.

We showed previously that EGFR phosphorylates human PIPKIγi2 at Y639 (Y634 in mouse PIPKIγ), which is critical for PIPKIγ function downstream of EGF [6]. This raises a possibility that loss of PIPKIγi2 disconnects activated EGFR from its downstream signaling cascades. To test this hypothesis, we re-expressed the wild type (WT) or the mutated (Y634F) mouse PIPKIγi2 (mPIPKIγi2) in L3.6 cells in which endogenous human PIPKIγ (hPIPKIγ) isoforms had been knocked down by specific shRNA (Figure 7A). These cells, stably expressing mPIPKIγi2-WT or mPIPKIγi2-Y634F and lack of endogenous hPIPKIγ, were then submitted to variant *in vitro* and *in vivo* analyses and compared to the pan-hPIPKIγ depleted cells.

As shown in Figure 7B, depletion of endogenous hPIPKIγ isoforms led to the similar decrease of cell proliferation in cells expressing empty vector, mPIPKIγi2-WT, or mPIPKIγi2-Y634F. This indicates that PIPKIγ isoforms likely function redundantly in supporting cell proliferation and restoration of PIPKIγi2 alone is not sufficient to recover cell proliferation defect caused by depleting all PIPKIγ isoforms in *in vitro* condition. On the other hand, cells expressing mPIPKIγi2-WT showed comparable EGF-induced cell migration with or without endogenous hPIPKIγ isoforms depleted, indicating that PIPKIγi2 is the PIPKIγ isoform responsible for directional cell migration responding to growth factors. However, mPIPKIγi2-Y634F failed to do so (Figure 7C), suggesting that EGFR-mediated PIPKIγi2 phosphorylation is critical for induced directional cell migration. Moreover, FA assembly in mPIPKIγi2-WT expressing cells was not affected by pan-hPIPKIγ depletion, but cells expressing the empty vector or mPIPKIγi2-Y634F showed inhibition in FA assembly when endogenous hPIPKIγ isoforms were knocked down (Supplementary Figure 5 and Figure 7D). Similarly, expression of the wild type mPIPKIγi2, but not the Y634F mutant, restored the matrix degradation that was inhibited by pan-hPIPKIγ depletion (Supplementary Figure 6 and Figure 7E). These results emphasized the importance of PIPKIγi2 in migration and invasion of PDAC cells, which are critical cellular processes for the development of tumor metastasis.

**Figure 8:**
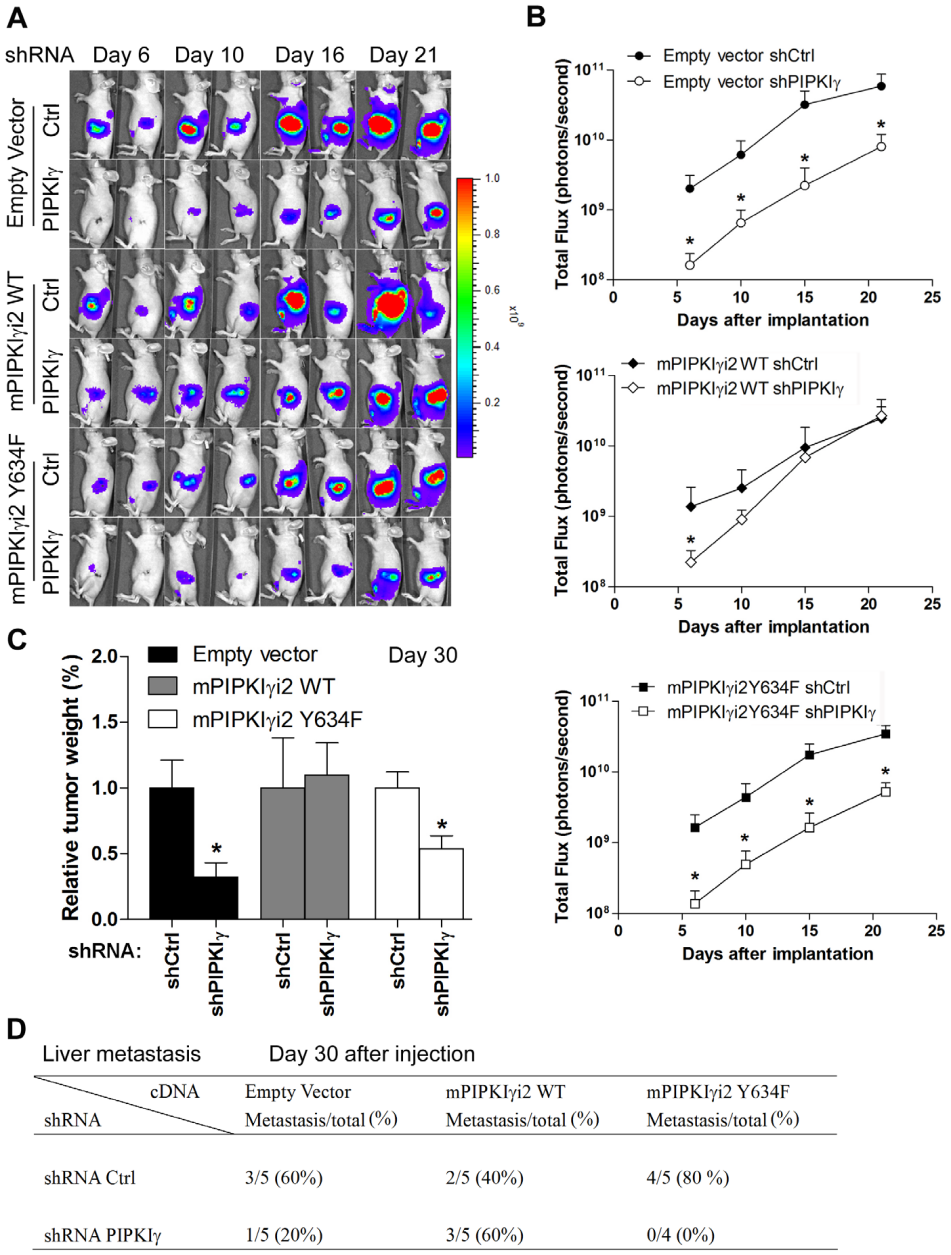
PIPKIγi2, functioning downstream of EGFR, promotes the progression of PDAC tumor. L3.6 cells were infected with lentivirus carrying luciferase expression cassette and then transduced with lentivirus carrying expression cassette of wild type (mPIPKIγ-WT) or mutated (mPIPKIγ-Y634F) to achieve the stable expression of luciferase with or without co-expression of mPIPKIγ-WT or mPIPKIγ-Y634F. Each of these lines were then infected with lentivirus carrying shRNA expression cassette of control or pan-hPIPKIγ shRNA, creating six luciferase-positive cell lines with endogeneous hPIPKIγ isoform depleted or not and exogenous mPIPKIγ expressed or not. These six lines were then inoculated to pancreas of nude mice (5 animals/group) via surgery to observe tumor progression, respectively. Expression of wild type mPIPKIγ, but not the EGFR-phosphorylation deficient mutant, recovered tumor growth **(A-C)** and liver metastasis **(D)**. **(A)** Representative luminescence images of nude mice injected with control and PIPKIγ depletion were shown at indicated time points after orthotopic transplant. **(B)** The values of photons/s/cm2/steridian depicting bioluminescence area in different groups were calculated and plotted. **(C and D)** Mice in all six groups were sacrificed at day 30 after injection. Then the weight of pancreas was measured and plotted **(C)** and number of animals with liver metastasis in each group was summarized **(D)**. *, *p*<0.05.

In the context that depletion of all PIPKIγ isoforms resulted in diminished metastasis in orthotopic PDAC mouse model (Figure 6), we proposed that PIPKIγi2 is the major contributor to PDAC progression *in vivo*. To test this, we introduced the stable expression of luciferase in each of the follwoing six L3.6 cell lines stably expressing (1) control vector, (2) control vector plus pan-hPIPKIγ shRNA, (3) mPIPKIγi2-WT, (4) mPIPKIγi2-WT plus pan-hPIPKIγ shRNA, (5) mPIPKIγi2-Y634F, and (6) mPIPKIγi2-Y634F plus pan-hPIPKIγ shRNA, respectively. These cell lines were then used to create the orthotopic PDAC mouse models to study PDAC tumor progression as described above. With the representative luminescence images (day 6, 10, 16 and 21 post injection) shown in Figure 8A, data from all mice in different groups were quantified and plotted in Figure 8B. Consistent with our previous results (Figure 6C), mice injected with pan-hPIPKIγ-depleted L3.6 cells showed markedly reduced tumor growth comparing to animals in the control group. Mice inoculated with cells expressing exogenous mPIPKIγi2-WT or mPIPKIγi2-Y634F exhibited similar tumor growth as ones inoculated with regular L3.6 cells. However, the expression of mPIPKIγi2-WT indeed protected tumor growth against the depletion of endogenous hPIPKIγ isoforms (Figure 8A-8C), although it did not efficiently recover *in vitro* cell proliferation measured by MTT assay (Figure 7B). We reason that the *in vivo* tumor growth does not only depend upon MAPK and AKT pathways. Other PIPKIγi2-dependent survival and growth pathways stimulated by the interaction with the tumor-associated microenvironment could compensate MAPK and AKT in promoting tumor progression. As shown in Figure 8B, the mPIPKIγi2-WT^+^/pan-hPIPKIγ^low^ cells exhibited slower growth rate at the early time points, but caught up quickly and reached the similar level as the control tumors when we sacrificed the animals at the end point of day-30 post inoculation (Figure 8C). However, mPIPKIγi2-Y634 failed to rescue the tumor growth defect caused by depleting endogenous hPIPKIγ at any tested time points (Figure 8A-8C).

At day-30 post inoculation, we examined the tumor metastasis in these mice. As summarized in Figure 8D, expression of mPIPKIγi2-WT completely abolished the reduction of liver metastasis caused by pan-hPIPKIγ depletion; however, expression of mPIPKIγi2-Y634F did not have such an effect. This observation is consistent with our results obtained from *in vitro* cell adhesion, migration and invasion assays (Figure 7C-7E), and further confirmed that PIPKIγi2, upon phosphorylated by EGFR at Y634, plays a key role in the development of PDAC metastasis.

## DISCUSSION

It is reported recently that activation of EGFR in pancreatic acinar cells is essential for the development of acinar-to-ductal metaplasia (ADM) and pancreatic tumorigenesis induced by constitutively activated KRAS [3]. As a unique process in the pancreas since KRAS dependent tumor development in lung and colon do not rely on EGFR signaling [32], EGFR activation synergistically supports the robust MEK/ERK and KRAS activities that are necessary for ADM formation but cannot be achieved solely by a single mutated *Kras* allele [3]. The requirement of EGFR activation for the very initial steps in pancreatic carcinogenesis opens the door for preventive approaches targeting EGFR signaling in patients at high risk of developing PDAC. Given that, only a subgroup of PDAC patients benefits from an EGFR-targeted therapy [9] and predictive biomarkers that determine benefit from erlotinib treatment have not yet been defined. This calls for the in-depth understanding of signaling cascades downstream of EGFR in the context of PDAC development and progression. In this study, we demonstrated that PIPKIγ is a key player downstream of EGFR activation in human PDAC and is required for PDAC progression. Examination of PDAC patient samples has shown upregulated EGFR (ERBB1) [33] in up to 90% of pancreatic tumors [34]. Consistently, we detected rapid phosphorylation of PIPKIγ at Y639 induced by EGF treatment in pancreatic cancer cells. More importantly, phosphorylated PIPKIγ highly presented in a large fraction of tumor tissues from PDA patients, preferentially in the tumors with lymph node metastasis. This is consistent with the high rate of abnormal elevation of EGFR in PDAC and suggests that PIPKIγ functions downstream of EGF signaling in promoting PDAC progression.

Activated EGFR transmits intracellular signals through many signaling cascades, including RAS/RAF/MAPK, PI3K/AKT, and STAT that regulate multiple cellular processes such as the proliferation, differentiation, migration, invasion, and survival of tumor cells. These cellular processes are responsible for all the important aspects of the development and progression of cancers including PDAC, therefore abnormally hyper-activated MAPK and AKT pathways by elevated EGF signaling are often observed in PDAC [35, 36]. Notably, STAT3 activation, which can also be induced by EGF stimulation [37], has been established as a mechanistic link between inflammatory damage and initiation of PDAC [38, 39]. Elevated STAT3 activity is associated with poor prognosis in patients with early stage PDAC undergoing pancreatic resection [40]. Our results demonstrate that depletion of PIPKIγ isoforms inhibits the EGF-induced activation of AKT, MAPKs and STAT3 in PDAC cells. This changes the *in vitro* behaviors of PDAC cells in proliferation, migration and invasion in response to EGF stimuli and subsequently leads to the inhibition of the *in vivo* growth and metastasis of PDAC tumors. These results indicate that PIPKIγ-defective PDAC tumors might benefit much less from upregulated EGF stimuli from the microenvironment and less resistant to nutrition deficiencies that often accompany with the rapidly growing tumor masses. PDAC is an infiltrating malignancy of the exocrine pancreas that features a robust and heterogeneous desmoplastic stromal response. A recent study suggested that STAT3 supports the expression of a drug-metabolizing enzyme and certain components of tumor-associated matrix that contribute to poor drug efficacy and delivery, therefore might also contribute to resistance to cytotoxic chemotherapy reported in PDAC [41]. In this context, our observations of PIPKIγ-dependent MAPK/AKT/STAT3 activation suggest an intriguing possibility that inhibiting PIPKIγ could serve as an important component of combination therapies by targeting both PDAC and PDAC-associated stroma.

In human cells, five splice variants of PIPKIγ have been reported. Among them, PIPKIγi1 is required for calcium signaling [42]; whereas PIPKIγi2 regulate FA dynamics, EGF-stimulated directional migration, basolateral targeting of E-cadherin, and endocytosis of the transferring receptor [7, 9, 43–47]. PIPKIγi3 was recently shown in regulating centriole duplication [48] and PIPKIγi5 regulates the endosomal trafficking and degradation of E-cadherin [49] and EGFR [50]. In addition, it has been reported recently that PIPKIγ is required for the activation of PI3K [51] that is critical for the survival and progression of PDAC. Our results confirmed that the EGF-mediated phosphorylation of PIPKIγi2 is required for the dissemination of PDAC cells to distant organs, however does not affect tumor growth. This is consistent with the defined roles of this particular PIPKIγ isoform in regulating cell adhesion and migration. Treatment of pan-PIPKIγ RNAi, which deplete all PIPKIγ isoforms, exhibited stronger inhibition on the progression of PDAC *in vivo*. These results indicate that different splicing isoform of PIPKIγ has both distinct and overlapping subcellular functions. Nevertheless, results from our current study indicate that inhibiting PIPKIγ could block the EGF signaling-dependent progression of PDAC.

In summary, we showed here that PIPKIγ depletion change the aggressive behavior of pancreatic cancer cells by regulating critical cellular events including proliferation, migration and invasion, which explained the molecular mechanism in dampening tumor growth and metastasis *in vivo*. Moreover, phosphorylated-PIPKIγ is highly expressed in human pancreatic tumors and metastatic lesions. This indicates that Y639 phosphorylation level of PIPKIγ could correlates with clinical characteristics of PDAC and should be further examined. Our new findings warrant further investigation to the therapeutic inhibition of PIPKIγ in PDAC and help reveal the molecular secrets of the regulation of EGFR-dependent signaling in this devastating disease. Understanding the precise role of PIPKIγ in pancreatic cancer and its cooperation with EGF signaling in the context of PDAC may enable development of ultimately better therapeutic strategies via suppression of PIPKIγ in combinaton with the use of EGFR inhibitors, tailored to the appropriate PDAC patient population.

## MATERIALS AND METHODS

### Cells, plasmids, lentivirus, and siRNAs

Human PDAC cell lines (L3.6, BxPC3, and DanG) were kindly provided by Dr. Mark McNiven (Mayo clinic, Rochester, MN). L3.6 cells were grown in MEM medium supplemented with 10% fetal bovine serum (FBS), essential vitamins, l-glutamine, sodium pyruvate, and streptomycin/penicillin. BxPC3 and DanG cells were maintained in RPMI 1640 or DMEM medium supplemented with 10% FBS, 100 U/ml penicillin and 100 μg/ml streptomycin.

Vectors encoding short hairpin RNA (shRNA) were constructed by cloning RNA oligonucleotides (Invitrogen, Carlsbad, CA) into pLKO.1 vector (AddGene, Cambridge, MA). The shRNA sequences targeting human pan-PIPKIγ and PIPKIγ isoform 2 (PIPKIγi2) were 5′-GTCGTGGTCATGAACAACA-3′ and 5′-GACGGCGAGAGCGACACATAA-3′. For lentivirus production, 293T cells were co-transfected with pLKO.1 carrying shRNA sequence and the packaging plasmids pCMV-ΔR8.91 and pMDG-VSV-G at a ratio of 2:1:1 using FuGENE® 6 transfection Reagent (Promega, Madison, WI). For protein overexpression, complementary DNA sequence of mouse PIPKIγi2 or PIPKIγi2-Y634F was cloned in the pCDH plasmid and lentivirus particles were produced in 293T cells by co-transfection of pCDH, pCMV-ΔR8.91 and pMDG-VSV-G at a ratio of 3:1:3. Medium containing viruse was collected 72 hours after transfection. To generate cells stably expressing shRNA, cells were selected using puromycin (5μg/ml) 36 hours post-transfection.

All small interfering RNA (siRNA) oligonucleotides were obtained from Invitrogen. The siRNA sequences targeting all human PIPKIγ isoforms (pan-PIPKIγ) are 5′-GTCGTGGTCATGAACAACA-3′ (PIPKIγ-1), and 5′- GCGTGGTCAAGATGCACCTCAAGTT-3′ (PIPKIγ-2). The siRNA sequences targeting human PIPKIγi2 is 5′- GAGCGACACAUAAUUUCUA -3′. Stealth RNAi Negative Control siRNA (Invitrogen, Carlsbad, CA) was used as negative control. siRNAs were introduced into cells using RNAiMAX (Invitrogen).

### Antibodies and reagents

Rabbit polyclonal polyclonal antibodies recognizing pan-PIPKIγ or Y639-phosphorylated PIPKIγ were generated as described previously [5]. HA antibody is from Millipore. Antibodies against phosphorylated ERK1/2, total ERK1/2, phosphorylated Akt, total Akt, phosphorylated STAT3, total STAT3 are from Cell Signaling. β-actin and talin antibodies are from Sigma. Phospho-tyrosine antibody is from BD Biosciences. Alexa Flour 555-conjugated phalloidin, Alexa Flour 488-conjugated goat anti-mouse antibody, Alexa Flour 555-conjugated goat anti-rabbit antibody and Alexa Fluor 555-conjugated goat anti-rat antibody are from Molecular probes.

### *In vitro* cell proliferation and soft agar colony formation assay

For cell prolifereation assay, cells were seeded into 96-well plates at a density of 5 × 10^3^/well and cultured in tissue culture incubator for variant amount of time, then incubated with 3-(4,5-dimethylthiazol-2-yl)-2,5-diphenyltetrazolium bromide (MTT) solution (Millipore, Billerica, MA) for 4 hours at 37 °C. Color development solution was then added and mixed thoroughly. Cell viability was determined in a spectrophotometer by measuring the absorbance at 570 nm, with 630 nm as the reference wavelength. Every time point/cell sample was carried out in triplicates.

Soft agar assays were performed in cell culture medium containing 10% FCS using 6-well plates in triplicate. Cells (5×103) were mixed with 0.3% agar and plated on a layer of 0.6% agar in 6-well plates. Cells were incubated at 37°C for 14 days. Colonies were stained with MTT and scanned with Gelcount colony counter. Numbers of colonies were quantified using GelCount software. The experiment was repeated three times independently.

### Cell migration, matrix degradation, and invasion assays

The directional migration assays were performed using modified Boyden chamber (Neuroprobe, Gaithersburg, MD, USA) as described previously [5]. Serum-starved cells in the upper compartment were allowed to migrate at 37°C for 4-6 hours. Wound healing assays were performed as described [5]. Phase contrast images of the wound area were acquired with a 10× objective at 0 and 16 hours after cells were scratched. The width of the wound on each sample was determined by NIH ImageJ.

Matrix degradation was performed using coverslips coated with fluorescent gelatin. 50 μg/ml poly-L-lysine was used to pre-coat coverslips for 20 minutes at room temperature, washed with PBS, and fixed with ice-cold 0.5% glutaraldehyde for 15 minutes followed by extensive washing. Then coverslips were then inverted on an 80-μL drop of fluorescent gelatin matrix (0.2% gelatin and Alexa Fluor 488 gelatin at an 8:1 ratio) and incubated for 15 minutes at room temperature. Coverslips were washed with PBS and the residual reactive groups in the gelatin matrix were quenched with 5 mg/mL sodium borohydride in PBS for 10 minutes followed by further washing in PBS. 1.0×10^5^ cells were plated on the coated coverslips and incubated at 37°C for 6-8 hours. Degradation area was calculated as the total area covered by degradation holes/field in threshold images using the Analyze Particles tool in ImageJ software. The quantified area was then normalized against the numbers of cells. The invasive capability of cells was determined by using 24-well matrigel-coated transwell chambers with 8-μm pore size (BD Biosciences, Bedford, MA) as described [5]. 5×10^4^ cells were added to the inserts of Transwell chamber. After 20 hours of incubation in tissue culture incubator, invaded cells adhering on the lower surface of the membrane were fixed by 3.7% paraformaldehyde and stained with 0.2% crystal violet. The invasion index was determined following the manufacturer’s instruction.

### Xenograft PDAC mouse model

Female athymic nude mice, 4 to 5weeks old, were purchased from Harlan. Mice were maintained under specific pathogen-free environment under controlled conditions of light and humidity and received food and water. All animal studies were carried out according to the guidelines and experimental protocols approved by the Animal Care Committee of the Mayo Clinic, Rochester MN.

Mice were anesthetized with katamine/xylazine by intraperitoneal injection. A small left abdominal flank incision was made and the spleen exteriorized. Tumor cells (5 × 10^5^ in 30 μL PBS) were injected subcapsularly into the pancreas using a 30-gauge needle. A successful subcapsular intrapancreatic injection of tumor cells was identified by the appearance of a fluid bleb without leakage. To prevent leakage, a cotton swab was held for over the site of injection for 1 min. Mice were administered buprenorphine after surgery to minimize animal discomfort. Tumor growth was monitored weekly by bioluminesence imaging. Briefly, mice received intraperitoneal injection of 200μl D-Luciferin (15 mg/ml in PBS) (Glod Biotechnology, St Louis, MO), then anesthetized with isoflurane and imaged using a bioluminescence imaging (Caliper Life Sciences-Xenogen, Hopkinton, MA). The signal intensity was quantified as sum of all detected photons within the region of interest per second using Living Image software.

### Immunoblotting, immunofluorescence microscopy, and immunohistochemistry

Western blotting was carried out according to standard methods as described previously [9]. Cells cultured on collagen-coated coverslips were fixed in 3.7% paraformaldehyde for 10 minutes and permeablized with 0.2% Triton X-100 in PBS for 5 minutes. The non-specific binding was blocked by incubation of the coverslips with 3% bovine serum albumin (BSA) in PBS, and this was followed by incubation with primary antibodies in 3% BSA/PBS. After extensive washing with PBS, cells were incubated with appropriate Alexa-conjugated secondary antibodies. To visualize F-actin structures and nuclei, the cells were stained with Alexa-conjugated phalloidin and DAPI (4’,6-diamidino-2-phenylindole, dihydrochloride). The slides were examined under a Nikon Eclipse fluorescence microscope, and the images were captured and analyzed using ImageJ software.

Tissue sections (5μm) were cut from the recipient blocks and deparaffinized in xylene and rehydrated through alcohol gradient. The tissue sections were incubated with hydrogen peroxide (Dako, Carpenteria, CA) for 10 minutes to quench endogenous peroxidase activity. After that, sections were rinsed with PBS for three times. Antigen retrieval was performed using a steamer for 30 minutes in 0.1 M citrate buffer (pH 6.0). Serum free protein block (Dako, Carpenteria, CA) were used to prevent non-specific protein binding. Sections were then incubated with primary antibodies overnight at 4°C and Dako Envision anti-rabbit secondary antibody was applied at room temperature for 60 minutes. The slides were developed with DAB Peroxidase Substrate Kit (Dako, Carpenteria, CA). Slides were then counterstained with hematoxylin, dehydrated in ethanol, cleared in xylene and mounted with Permount mounting medium.

### Statistical analyses

Data are presented as mean ± standard error of the mean (SEM). Statistical analysis was carried out using Student's *t* tests. Two-tailed *P* values < 0.05 were considered statistically significant. Survival of tumor-bearing mice was estimated using Kaplan–Maier curve. Log-rank test was used to estimate the survival differences.

## SUPPLEMENTARY MATERIALS


