# Supplementary material for: EGFR-induced phosphorylation of type Iγ phosphatidylinositol phosphate kinase promotes pancreatic cancer progression
Source: Oncotarget. 2017 Mar 31;8(26):42621–37. doi: 10.18632/oncotarget.16730 (PMC5522093; doi:10.18632/oncotarget.16730)
Supplement: Supplementary file 1 [file oncotarget-08-42621-s001.pdf]

EGFR-induced phosphorylation of type Iγ phosphatidylinositol phosphate kinase promotes pancreatic cancer progression

SUPPLEMENTARY FIGURES

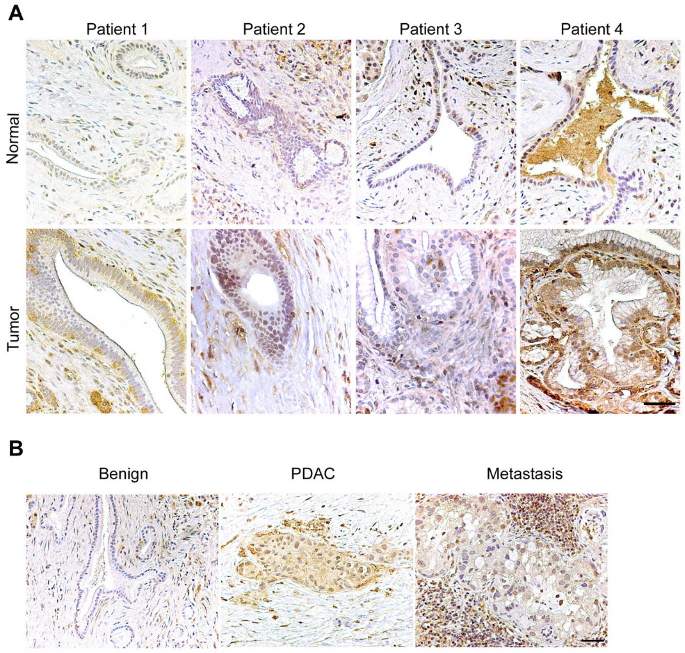

Supplementary Figure 1: Total protein levels of PIPKIγ in pancreatic tumors are not changed comparing to normal pancreatic tissues. Human PDAC patient samples corresponding to Figure 1C **(A)** and Figure 1D **(B)** were subjected to immunohistochemisrty staining using antibodies recognizing all PIPKIγ isoforms. Scale bar: 50μm.

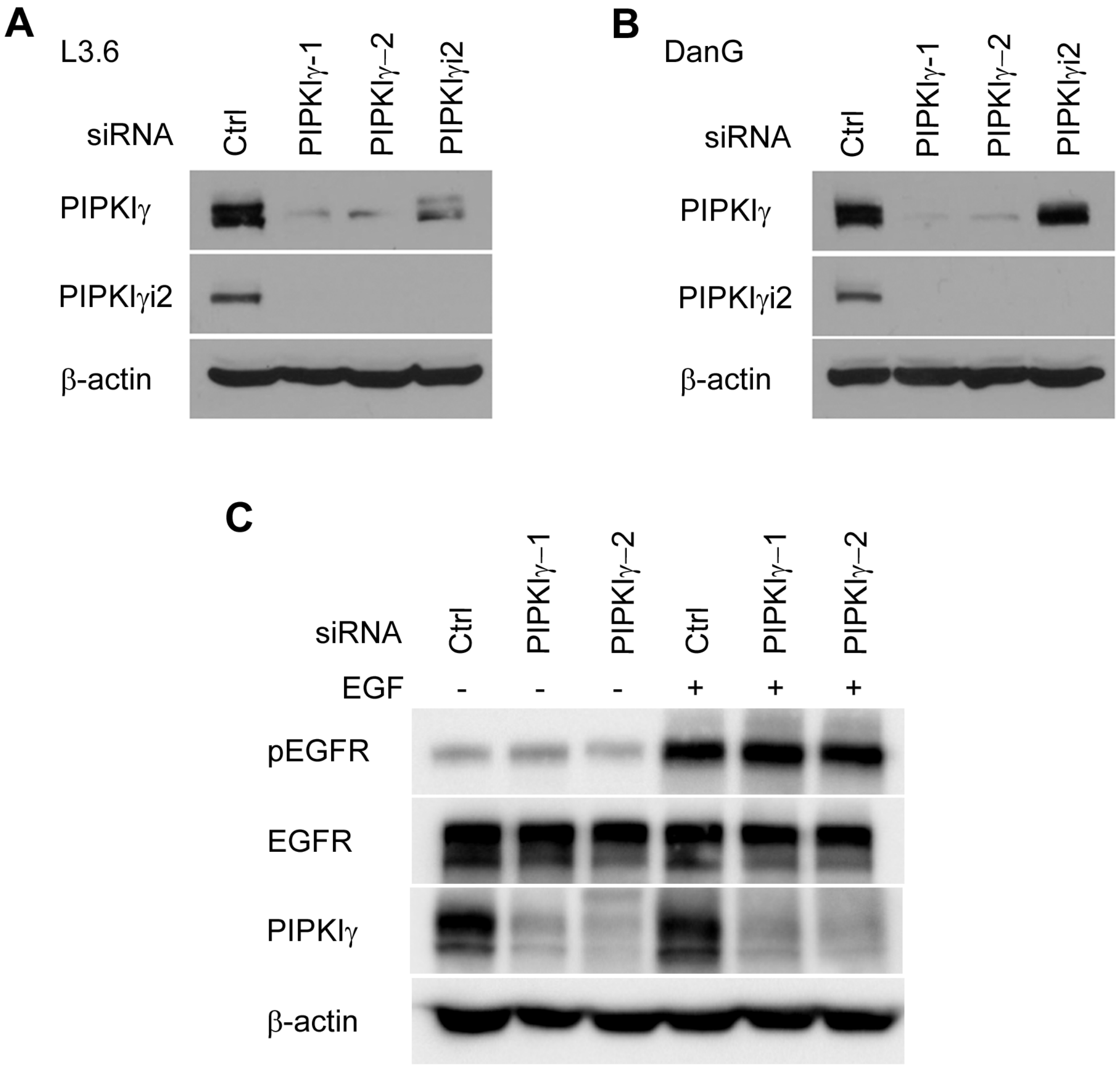

Supplementary Figure 2: Loss of PIPKIγ has no effect on EGFR in PDAC cells. (A and B) L3.6 or DanG cells were transfected with control, pan-PIPKIγ, or PIPKIγi2 specific siRNAs for 48 hours, and the knockdown efficiency of PIPKIγ or PIPKIγi2 siRNA was confirmed by immunoblotting. **(C)** L3.6 cells, 48-hrs post transfection with indicated siRNAs, were serum-starved overnight, treated with 10 ng/mL EGF for 15 minutes, and then analyzed by immunoblotting with indicated antibodies.

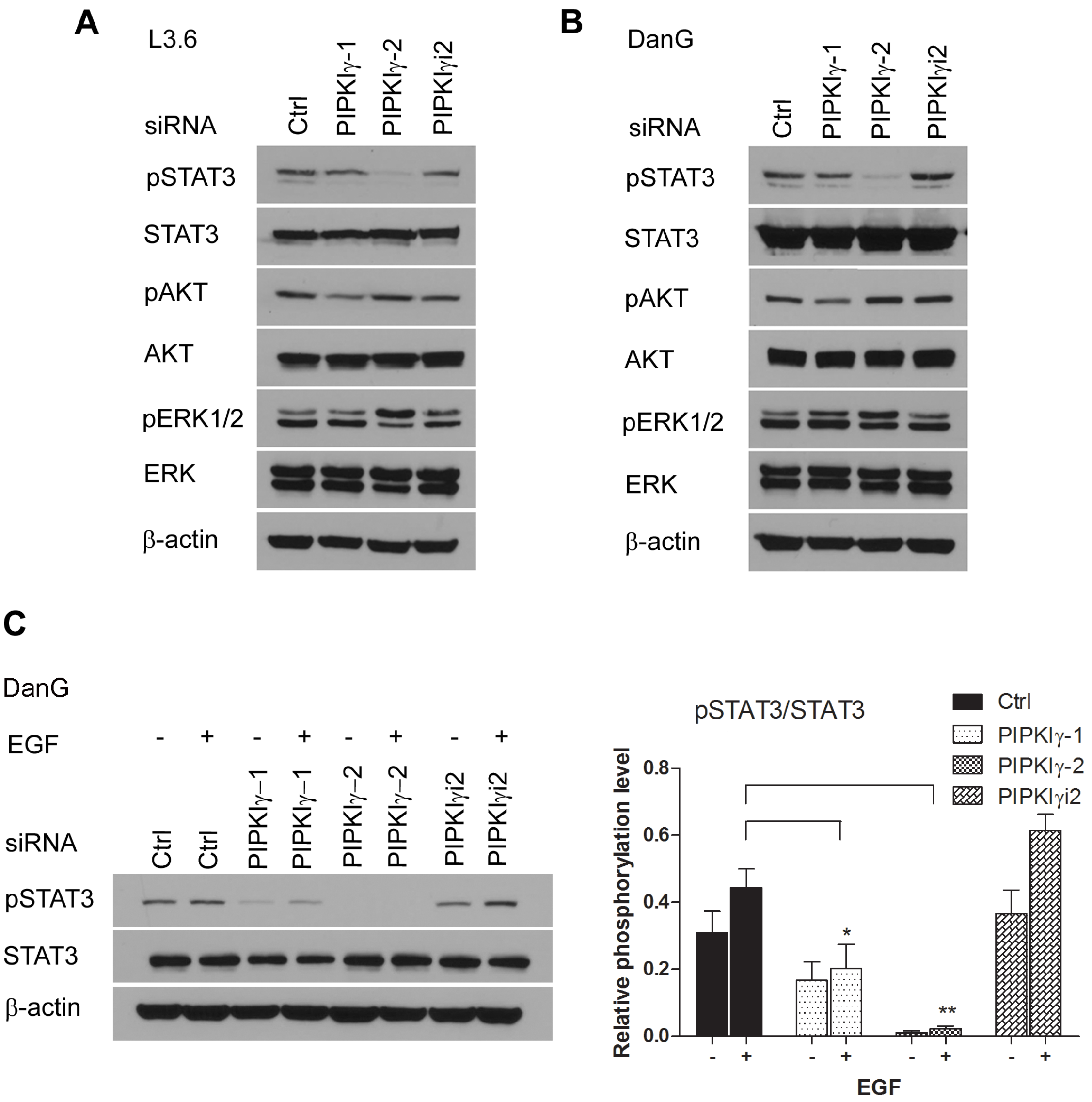

Supplementary Figure 3: Loss of PIPKIγ inhibits the activation of STAT3 in PDAC cells. (A and B) L3.6 or DanG cells were transfected with control, pan-PIPKIγ, or PIPKIγi2 specific siRNAs for 48 hours, and then subjected to immunoblotting with indicated antibodies to determine the activation of STAT3, AKT, or ERK1/2. **(C)** DanG cells, 48-hrs post transfection with indicated siRNAs, were serum-starved overnight, treated with 10 ng/mL EGF for 15 minutes, and then analyzed by immunoblotting with antibodies against phosphorylated STAT3 (pSTAT3) and total STAT3 as well as actin. Right panel, the level of activated STAT3 in each group was quantified and plotted. The intensities of pSTAT3 and STAT3 bands were quantified using ImageJ. STAT3 activation was represented by the ratio of pSTAT3 and STAT3. Results from three independent experiments were statistically analyzed and plotted. *, $p < 0.05$; ** $p < 0.01$.

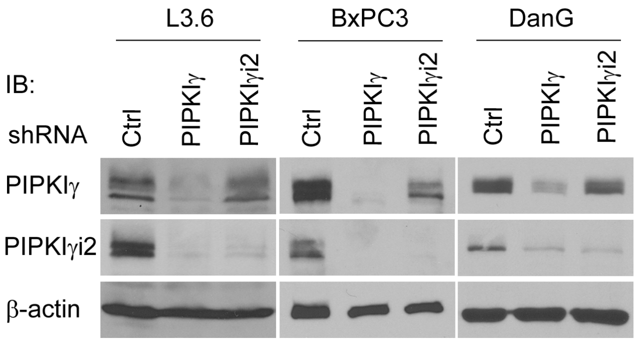

Supplementary Figure 4: Lentivirus-mediated expression of pan-PIPKIγ or PIPKIγi2 shRNAs efficiently knocked down all PIPKIγ isoforms or PIPKIγi2 from PDAC cells, respectively. Lentivirus particles carrying expression cassette of control, pan-PIPKIγ, or PIPKIγi2 shRNA were incubated with indicated cells for 48 hours. Cells were then analyzed by immunoblotting using indicated antibodies.

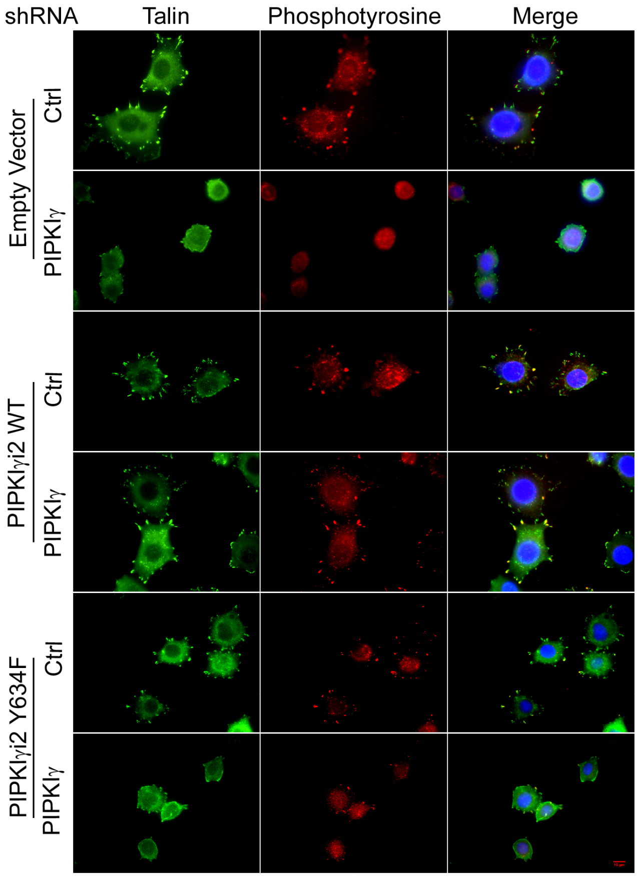

Supplementary Figure 5: Y634 phosphorylation in PIPKIγi2 is required to focal adhesion assembly in PDAC cells. L3.6 cells stably expressing empty vector, wild type (mPIPKIγ-WT), or Y634F (mPIPKIγ-Y634F) mouse PIPKIγ were infected with lentivirus carrying indicated shRNA for 48 hours. These cells were re-plated on collagen-coated coverslips for 1 hour, processed through indirect immunofluorescence staining to visualize talin and phosphotyrosine with nuclei labeled by DAPI. Cells were then imaged using fluorescence microscope to examine the formation of focal adhesions. Scale bar, 10μm.

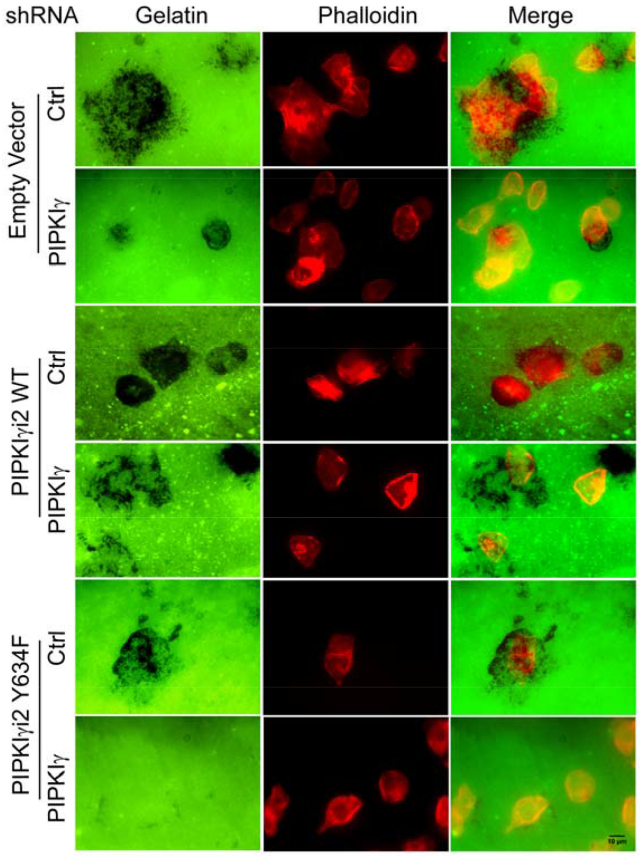

Supplementary Figure 6: Wild type, but not the EGFR-phosphorylation deficient, PIPKIγi2 rescues the reduced matrix degradation in PIPKIγ-depleted DanG cells. Parental (control) or PIPKIγ-depleted (PIPKIγ) DanG cells transfected with empty vector, mPIPKIγ WT, mPIPKIγ Y634F. Cells were plated on fluorescent gelatin-coated coverslips (green) for 4 hrs, then fixed and stained with phalloidin (red). Representative images were then taken under fluorescence microscope. Expression of the wild type, but not the Y634-mutated, mPIPKIγi2 can rescue the defective focal adhesion assembly caused by depletion of endogenous PIPKIγ. Scale bar, 10 µm.
